# Occupational Carcinogens: ELF MFs

**DOI:** 10.1289/ehp.113-1310936

**Published:** 2005-11

**Authors:** Kjell Hanson Mild, Mats-Olof Mattsso, Lennart Hardell, Joseph D. Bowman, Michael Kundi

**Affiliations:** National Institute for Working Life, Umeå, Sweden; Örebro University, Örebro, Sweden; National Institute for Occupational, Safety and Health, Cincinnati, Ohio, E-mail: jdb0@cdc.gov; Medical University of Vienna, Vienna, Austria

Siemiatycki et al. (2004) published a list of occupational carcinogens based largely on the evaluations published by the International Agency for Research on Cancer (IARC), augmented with additional information on the extent of workplace exposure. They considered 28 agents as definite human occupational carcinogens (IARC group 1), 27 agents as probable occupational carcinogens (group 2A), and 113 agents as possible occupational carcinogens (group 2B). However, missing from their list of occupational carcinogens is magnetic fields (MFs) at extremely low frequencies (ELF; 3–3000-Hz), which were classified as group 2B by IARC (2002).

IARC’s final conclusion (IARC 2002) is as follows:

Overall, extremely low frequency magnetic fields were evaluated as possibly carcinogenic to humans (IIB), based on the statistical association of higher level residential ELF magnetic fields and increased risk for childhood leukaemia.

Thus, although the evaluation is based on epidemiologic studies of childhood leukemia, the classification applies to all human exposure to ELF MFs, and thus also to occupational exposure. This interpretation has been discussed and confirmed with an IARC representative on their ELF MF panel (Cardis E, personal communication). Because enough workers are exposed to ELF MFs to clearly meet the criteria for occupational exposures set by Siemiatycki et al. (2004), we are surprised that they did not include it in their list of possible occupational carcinogens.

Other groups and agencies have applied IARC’s criteria to the evaluation of ELF MF carcinogenicity. The National Institute of Environmental Health Sciences working group (NIEHS 1998) evaluated the research in that era and classified ELF EMFs (electric and magnetic fields) as possibly carcinogenic (group 2B); this classification was based on the occurrence of chronic lymphocytic leukemia (CLL) associated with occupational exposure. The California Department of Health Services also evaluated the cancer risks of EMF in 2002, and their reviewers classified it as at least group 2B, including childhood leukemia and adult brain cancer (Neutra et al. 2002).

Since the IARC evaluation, several relevant studies have been published—both *in vitro* and *in vivo* work, as well as epidemiologic studies, including the following examples. Tynes et al. (2003) reported an association between exposure to calculated residential MFs and cutaneous malignant melanoma. In a cohort including all female workers, Weiderpass et al. (2003) found an association between exposure to electromagnetic fields and stomach and pancreatic cancer; Villeneuve et al. (2002) found that occupational MF exposure increased the risk of glioblastoma multiforme; Håkansson et al. (2002) investigated cancer incidence in resistance welding workers exposed to high levels of MF and found that men in the very high exposure group showed an increased incidence of tumors of the kidney, pituitary gland, biliary passages, and liver; an exposure–response relationship was indicated for these cancer sites. Women in the very high exposure group showed an increased incidence of astrocytoma I–IV, with a clear exposure–response pattern.

Ivancsits et al. (2002, 2003a, 2003b) have shown that human lymphocytes exposed to ELF MFs can generate DNA single and double strand breaks from a flux density as low as 35 μT and with a strong correlation between both the intensity and duration of the MF exposure.

The IARC evaluation (IARC 2002) ruled out a probable carcinogen classification (group 2A) because the expert panel found the animal studies were “inadequate evidence of carcinogenicity.” This judgment was due to many conflicting results in the repetition of long-term animal experiments. In particular, Löscher and Mevissen (1995) reported that MF exposure to Sprague-Dawley (SD) rats after 7,12-dimethylbenz[*a*]anthracene (DMBA) initiation increased breast tumors in the exposed animals at 50 μT compared with the control group (see also Thun-Battersby et al. 1999). However, in a similar study Anderson et al. (1999) found no evidence for a cocarcinogenic or tumor-promoting effect of MF exposure, but the study used different substrains of SD rats than used in the original study. Anderson et al. (2000) stated that “the U.S. rats were more susceptible to DMBA than the European rats”; diet and DMBA were from different sources, and there were differences in environmental conditions and in MF exposure metrics. Fedrowitz et al. (2004) compared two sub-strains of SD outbred rats; MF exposure significantly increased mammary tumor development and growth in one of the strains of rats but not in the other. These data suggest that genetic background may play a pivotal role in effects of MF exposure; this which might explain the difficulties in replicating the original animal studies of breast tumor promotion.

According to the criteria used by Siemiatycki et al. (2004), a complete list of occupational agents classified as possible human carcinogens would include ELF MFs.
